# Editorial: Rising stars in virus and host: 2022

**DOI:** 10.3389/fcimb.2022.1100695

**Published:** 2022-12-01

**Authors:** Andres López-Cortés, Santiago Guerrero

**Affiliations:** ^1^ Cancer Research Group (CRG), Faculty of Medicine, Universidad de Las Américas, Quito, Ecuador; ^2^ Latin American Network for the Implementation and Validation of Clinical Pharmacogenomics Guidelines (RELIVAF-CYTED), Madrid, Spain; ^3^ Laboratorio de Ciencia de Datos Biomédicos, Escuela de Medicina, Facultad de Ciencias Médicas de la Salud y de la Vida, Universidad Internacional del Ecuador, Quito, Ecuador

**Keywords:** SARS-CoV-2, COVID-19, reinfection, H7N9, leishmania, herpes simplex virus type 1, phage therapy, *Enterovirus A (normal)*

Recognizing the future leaders of virology is fundamental to safeguard tomorrow’s driving force in innovation for this fundamental research field. In this context, the objective of the Rising Stars in Virus and Host: 2022 article collection was to highlight high-quality research across the entire breadth of virology focusing on pending issues, new challenges, and future innovations. The research presented in this collection highlights worldwide effort to understand the complexity of virus concerning their infection mechanisms, immunological responses or even for phage-based therapies against multidrug-resistant bacteria.

Coronavirus disease 2019 (COVID-2019) and its causative agent, severe acute respiratory syndrome coronavirus 2 (SARS-CoV-2) continue to be the focus of intensive research. Indeed, this Research Topic contains three scientific articles related to multiple aspects of the disease. Firstly, Wang et al. have reviewed the immune responses of SARS-CoV-2 infection. This study summarizes the molecular underpinnings of innate immune mechanisms behind virus-host interactions, which can contribute to the development of effective therapeutic avenues. Secondly, Guevara et al. have provided epidemiological data concerning SARS-CoV-2 reinfection in Ecuador. Their study showed, by a 22 months-long surveillance strategy, that reinfection frequency has increased 10-fold following the introduction of the Omicron variant. Interestingly, this study performed the longest monitoring of reinfection events, showing an occurrence at regular intervals of 4-5 months, and confirming Omicron as the main cause of reinfection. Lastly, Wang et al. have discussed the current understanding of early differentiation of virus-specific effector follicular helper T-cells (TFH) and long-term maintenance of virus-specific memory cells in mouse models and patients with SARS-CoV-2 infection.

To shed light on the interaction between host immunity and influenza evasion, Zhu et al. studied the mechanisms underlying the virulence, pathogenicity, and immune response between H9N2 and H7N9 virus infections that cause influenza A. They used a mouse infection model to dissect the difference in the host response between both viruses through transcriptomic analyses of infected lungs. Consequently, they discovered that the H9N2-infected lungs elicited an earlier induction of innate immune response and earlier recruitment of macrophages than the H7N9-infected lungs. Finally, the different patterns of immune response may underlie more severe lung pathology caused by H7N9 infection compared to H9N2 infection.

Type I interferons (IFNs) are the first line of defense against viral infections, promoting antiviral, immunomodulatory, and antiproliferative effects. The IFN family contains 12 INF-α subtypes and IFN-β. However, the biological impact of individual subtypes remains controversial and drug-resistant infections with herpes simplex virus type 1 (HSV-1) are still lacking a protective or curing therapy in recurrent orogenital lesions. In this context, Schmitz et al. evaluated selective INF-α subtypes and IFN-β for their therapeutic potential in genital HSV-1 infections. Their results provided further insights into the diversity of IFN effector functions and their impact on the immunological control of HSV-1 infections.

Leishmania RNA virus 1 (LRV1) is a double-stranded RNA (dsRNA) virus found in some strains of the human protozoan parasite Leishmania, the causative agent of leishmaniasis, a neglected tropical disease. Jha et al. have explained the crucial role that the lymphatic system plays in infectious metastasis caused by Leishmania. Their results demonstrated that when infection in mucocutaneous leishmaniasis is associated with inflammation and LRV1, lymphatic vessels could serve as efficient routes of dissemination for infected cells to colonize distant organs from primary sites. On the other hand, the presence of LRV1 inside Leishmania constitutes an important virulence factor that worsens the leishmaniasis outcome in an IFN–dependent manner and contributes to treatment failure. Bekkar et al. contributed to shed light and dissect the intricate macrophage response toward infection by the Leishmania-LRV1 duo, providing new therapeutic strategies.

Bai et al. discovered promising prospects in the treatment of Carbapenem-Resistant *Klebsiella pneumoniae* (CRKP) infections by using phages and phage-encoded proteins. They isolated and identified a novel *Klebsiella pneumoniae* phage (vB_kpnM_17-11) using a CRKP host. vB_kpnM_17-11 belongs to the family of Myoviridae, order Caudovirales, and its genome is a double-stranded DNA (dsDNA) containing 165,894 base pairs and 275 Open Reading Frames (ORFs). Lastly, their results showed excellent *in vitro* and *in vivo* performance against *Klebsiella pneumoniae* infection and constitutes a promising candidate for the development of phage therapy against CRKP.

Enterovirus A (EV-A) species cause hand, foot, and mouth disease (HFMD) in young children. To better understand EV-A evolution and adaptation, Zeng et al. have examined multiple codon usage parameters finding that the codon usage bias among EV-A strains varies and is clade-specific. Additionally, they revealed that the codon usage patterns of EV-A strains were shaped by mutation pressure and natural selection. These findings have allowed them to identify novel characteristics of codon usage bias in distinct EV-A clades associated with their host range, transmission, and pathogenicity.

Finally, Chandra et al. have written an interesting review on the implications of host components in regulating integrity and dynamics of a dsRNA rotavirus (RV) replication factories. Their article focused on the interaction between the replication bodies (viroplasms) of a dsRNA RV and the host cellular determinants of infection to provide a platform for designing host-directed antiviral therapeutic interventions in the future.

## Author contributions

All authors confirm being contributors of this work and have approved it for publication.

## Conflict of interest

The authors declare that the research was conducted in the absence of any commercial or financial relationships that could be construed as a potential conflict of interest.

## Publisher’s note

All claims expressed in this article are solely those of the authors and do not necessarily represent those of their affiliated organizations, or those of the publisher, the editors and the reviewers. Any product that may be evaluated in this article, or claim that may be made by its manufacturer, is not guaranteed or endorsed by the publisher.

